# Everyday executive functions in Down syndrome from early childhood to young adulthood: evidence for both unique and shared characteristics compared to youth with sex chromosome trisomy (XXX and XXY)

**DOI:** 10.3389/fnbeh.2015.00264

**Published:** 2015-10-20

**Authors:** Nancy Raitano Lee, Payal Anand, Elizabeth Will, Elizabeth I. Adeyemi, Liv S. Clasen, Jonathan D. Blumenthal, Jay N. Giedd, Lisa A. Daunhauer, Deborah J. Fidler, Jamie O. Edgin

**Affiliations:** ^1^Child Psychiatry Branch, National Institute of Mental HealthBethesda, MD, USA; ^2^Department of Psychology, Drexel UniversityPhiladelphia, PA, USA; ^3^Department of Psychology, University of ArizonaTucson, AZ, USA; ^4^Human Development and Family Studies, Colorado State UniversityFort Collins, CO, USA; ^5^Department of Psychiatry, University of CaliforniaSan Diego, La Jolla, CA, USA

**Keywords:** executive function, age, development, Trisomy 21, klinefelter syndrome, trisomy X syndrome, behavior, aneuploidy

## Abstract

Executive functions (EF) are thought to be impaired in Down syndrome (DS) and sex chromosome trisomy (Klinefelter and Trisomy X syndromes; +1X). However, the syndromic specificity and developmental trajectories associated with EF difficulties in these groups are poorly understood. The current investigation (a) compared everyday EF difficulties in youth with DS, +1X, and typical development (TD); and (b) examined relations between age and EF difficulties in these two groups and a TD control group cross-sectionally. Study 1 investigated the syndromic specificity of EF profiles on the Behavior Rating Inventory of Executive Function (BRIEF) in DS (*n* = 30), +1X (*n* = 30), and a TD group (*n* = 30), ages 5–18 years. Study 2 examined age effects on EF in the same cross-sectional sample of participants included in Study 1. Study 3 sought to replicate Study 2's findings for DS by examining age-EF relations in a large independent sample of youth with DS (*n* = 85) and TD (*n* = 43), ages 4–24 years. Study 1 found evidence for both unique and shared EF impairments for the DS and +1X groups. Most notably, youth with +1X had relatively uniform EF impairments on the BRIEF scales, while the DS group showed an uneven BRIEF profile with relative strengths and weaknesses. Studies 2 and 3 provided support for fairly similar age-EF relations in the DS and TD groups. In contrast, for the +1X group, findings were mixed; 6 BRIEF scales showed similar age-EF relations to the TD group and 2 showed greater EF difficulties at older ages for +1X. These findings will be discussed within the context of efforts to identify syndrome specific cognitive-behavioral profiles for youth with different genetic syndromes in order to inform basic science investigations into the etiology of EF difficulties in these groups and to develop treatment approaches that are tailored to the needs of these groups.

## Introduction

Over the past several decades, a great deal of progress has been made in characterizing the behavioral phenotypes associated with different genetic disorders—from disorders characterized by small microdeletions to full chromosomal aneuploidies (see Waite et al., 2014 for a review). While increases in knowledge about different behavioral phenotypes have been substantial, additional research is needed to isolate syndrome-specific characteristics from characteristics that are shared across syndromes. In the current investigation, we compare executive function (EF) profiles in Down syndrome (DS), sex chromosome trisomy (Klinefelter and Trisomy X syndromes; +1X), and typical development (TD). By comparing EF profiles in youth with these chromosomal trisomies not only to that of TD youth but also to one another, we aim to identify *etiologically-specific* characteristics of DS and +1X that may serve as more specific intervention targets for psychosocial and biomedical interventions.

The current investigation utilizes a caregiver report measure of EF, the Behavior Rating Inventory of Executive Function (BRIEF), to quantify the nature and severity of everyday EF difficulties in youth with DS and +1X. The BRIEF is a widely used measure in studies of DS and other developmental disorders. It is a useful tool for characterizing EF difficulties, particularly in populations such as DS and +1X, where there is considerable variability in cognitive ability levels. This cognitive variability can sometimes preclude the use of traditional laboratory measures for *all* participants in a given study. Thus, caregiver report can serve as an important index of real-world function and quality of life for participants with DS and +1X with a large range of ability levels. This has particular relevance for clinical trials assessment, as it permits a standard metric that can be used to evaluate change in functioning for all participants regardless of cognitive ability. Here we present cross-sectional data on the BRIEF that reveals a specific profile of ability and developmental trajectory in DS and +1X. Given that this measure is currently in use in a number of DS clinical studies underway, these data could serve as a benchmark for future work with this group.

In the sections that follow, we will describe the cognitive construct of EF and summarize one theoretical model that conceptualizes relations between different EF abilities. Then we will summarize the literature on the neuropsychology of DS and +1X, with a particular focus on what is known about EF abilities in these two groups. We will conclude with a description of the current study's research questions and hypotheses.

EF is an umbrella term used to describe a collection of higher-level cognitive abilities thought to be important for completing goals. A number of different abilities have been ascribed to this umbrella term, including working memory, planning, inhibition, and cognitive flexibility (Miyake et al., 2000; Lezak et al., 2004). EF abilities are thought to be important for various real world outcomes, including academic achievement (Blair and Razza, 2007) and work behavior (Ready et al., 2001).

Different conceptualizations of EF abilities exist. One conceptualization emphasizes the distinction between more cognitively-dominated executive processes, called cool EF (thought to be related to the functioning of the dorsolateral prefrontal cortex) and more affectively-heavy executive processes, called hot EF [thought to be related to the functioning of ventromedial prefrontal cortex (Metcalfe and Mischel, 1999; Zelazo and Muller, 2002)]. Examples of cool EF laboratory tasks include working memory tasks, such as backward digit span or spatial working memory, and planning tasks, such as the Tower of London. Examples of hot EF laboratory tasks include delay of gratification tasks and the Iowa Gambling task, among others. These hot EF tasks are thought to invoke the so-called “reward system”—that is, they require individuals to make choices that impact the size and/or immediacy of receiving a reward. Thus, they implicate motivation and affective systems more than traditional cool EF tasks. It has been proposed that different developmental disorders may be characterized by different cool and hot EF profiles (Zelazo and Muller, 2002). Thus, in the current study we seek to examine similarities and differences in the profile of cool and hot executive abilities in youth with DS and those with an additional X chromosome. In the next sections, we will summarize what is known about EF abilities and neuropsychological functioning more generally in individuals with DS and those with +1X.

Individuals with DS most often have IQs in the range of intellectual disability (standard scores <70); however, the neuropsychological phenotype in DS is more specifically characterized by language deficits in articulation and syntax (see Fowler et al., 1994 for a review) along with profound weaknesses in verbal short-term/working memory (see Baddeley and Jarrold, 2007 for a review). Additionally, DS is characterized by weaknesses in associative memory as well as motor delays (Pennington et al., 2003; Vicari, 2006). In contrast, some aspects of visual-spatial abilities, particularly visual-spatial short-term memory, and implicit learning have been reported to be mental age appropriate (Silverstein et al., 1992; Wang and Bellugi, 1994; Vicari et al., 2007). Furthermore, research examining behavioral difficulties in DS documents lower rates of problems compared to peers with other forms of intellectual or developmental disability (Dykens, 2007; though rates are higher than TD peers of similar chronological age).

Most studies of EF abilities in DS have examined one EF domain (e.g., working memory, inhibition) or have focused exclusively on more traditional cool, cognitively-mediated EF abilities within the laboratory setting. With a few exceptions (e.g., Pennington et al., 2003), these studies have documented deficits in the EF domains of inhibition, planning and problem-solving, cognitive flexibility/set-shifting, and working memory relative to typically-developing children matched on mental age or children with other forms of intellectual disability (Lanfranchi et al., 2004; Rowe et al., 2006; Lanfranchi et al., 2010; for a review, see Lee et al., 2011a, **Table 2**).

In our prior work (Lee et al., 2011a) using the BRIEF—Preschool (BRIEF-P; Gioia et al., 2003), we found evidence for a specific DS profile relative to norms appropriate for mental-age. Specifically, this young sample of children with DS (mean age ~6 years) demonstrated greater deficits in the so-called “cool” executive functions, such as working memory and planning, than the so-called “hot” executive functions, such as behavioral inhibition and emotional control (which were found to be commensurate with mental-age expectations but below chronological-age expectations). A more recent investigation by our group (Daunhauer et al., 2014) in which youth with DS were compared to MA-matched typically developing controls revealed a similar profile—that is, greater “cool” than “hot” EF difficulties. However, this study also documented inhibition difficulties (according to parent, but not teacher report) that exceeded mental age expectations, suggesting that the domain of behavioral inhibition may need to be investigated further in this group.

To the best of our knowledge, no studies have examined relations between age and EF abilities in youth with DS. This is particularly important for DS, as it is a disorder that is characterized by a slowing of cognitive development beginning in infancy (Hodapp and Zigler, 1990; Carr, 2005) as well as precocious onset of Alzheimer's disease in the fifth to sixth decades of life (Lott, 1982). Further, EF abilities show early emerging decline in adulthood in DS (Ball et al., 2006, 2008). Consequently, understanding the developmental stability of EF will be important for understanding the unfolding of the DS cognitive phenotype from childhood to young adulthood as well as informing studies seeking to identify individuals with DS who are at greatest risk for developing Alzheimer's disease later in life. We now turn to the literature on the neuropsychology of +1X.

Unlike DS, neither Klinefelter nor Trisomy X syndrome are typically associated with intellectual disability. Rather research suggests that the presence of an additional X chromosome is associated with approximately one standard deviation reduction in intellectual abilities relative to siblings or a well-matched typically developing control group (Polani, 1977). However, similar to DS, high rates of language-based learning disorders occur, including articulation difficulties, deficits in syntax, verbal memory weaknesses, and reading difficulties (for reviews, see Leggett et al., 2010; Lee et al., 2011b). Reports of behavioral and psychiatric difficulties in females and males with supernumerary X chromosomes have identified heightened rates of depressive and anxiety disorders in females (see Tartaglia et al., 2010 for a review) and heightened rates of attention and social difficulties in males (Tartaglia et al., 2006; Bruining et al., 2010).

EF difficulties, particularly on tasks with pronounced verbal demands, have been well-documented in Klinefelter syndrome. Deficits have been reported on tasks of verbal inhibition and verbal working memory as well as verbal fluency, the Trail Making Test, and both spatial working memory and planning tasks, such as the Stockings of Cambridge (Bender et al., 1993; Ross et al., 2009; Van Rijn et al., 2009; Lee et al., 2011c). For females with Trisomy X, limited data exist on EF abilities. However, the few studies that have examined EF abilities have documented weaknesses on tasks including the Wisconsin Card Sorting task and verbal fluency. Additionally, there have been reports of reduced attentional abilities relative to either siblings or typically-developing control participants (Bender et al., 1993, 2001). To our knowledge, no published papers have examined everyday EF abilities in males and females with an additional X chromosome. Moreover, no studies have examined the relations between age and EF difficulties in youth with sex chromosome trisomies. Thus, the current study will be the first report of its kind. In the section that follows, we summarize the questions asked by this investigation and the study hypotheses.

In the current investigation, we sought to answer two questions: (1) Are there unique EF profiles on the BRIEF for school-age children and adolescents (ages 5–18) with DS and those with +1X? (2) Do the relations between age and EF abilities in DS and +1X deviate from what is seen in TD (in this cross-sectional sample)?

Regarding question 1, we tested two competing hypotheses. Hypothesis 1 posited that there would be specificity for the profile of EF difficulties associated with DS and +1X (i.e., these disorders would be characterized by different patterns of scores on the BRIEF). Hypothesis 2 posited non-specificity—that is, the disorders would have a similar profile of scores, such that the two disorders cannot be discriminated based on BRIEF scores alone.

Regarding question 2, we tested three competing hypotheses. The first predicted developmental stability in EF problem behaviors for the DS and +1X groups—that is, the extent to which individuals with DS or +1X have EF difficulties in everyday life will be similar in magnitude in early childhood and young adulthood. This finding would mirror the pattern found in TD and would suggest that EF difficulties are present from early in development (prior to the ages studied here) and that the magnitude of these difficulties persists across the developmental period studied. The second hypothesis predicted developmental variability—that is, deviations in EF skills in youth with DS or +1X will differ at different stages of development. This may be reflected in a lessening of difficulties from early childhood to young adulthood such that deviations from TD decrease. Such a finding would be consistent with studies of other developmental disorders, such as specific language impairment, in which some research suggests that behavioral difficulties lessen as children age (St Clair et al., 2011). Conversely, increasing EF difficulties relative to typical peers may become apparent with age. This latter scenario is similar to that reported for youth with autism on the BRIEF, in which difficulties on several scales were found to show increasing impairment with age (Rosenthal et al., 2013).

These questions were investigated in three studies. Study 1 investigated the syndromic specificity of EF profiles for DS and sex chromosome trisomy using a traditional case-control design. Study 2 investigated age-effects on EF profiles for these two groups and contrasted findings with TD youth. Study 3 sought to replicate the DS age-effect findings from study 2 by examining age-EF relations in a large independent sample of youth with DS and TD representing a larger age range than included in Study 2.

## Study 1: Contrasting the DS and +1X profile on the BRIEF

### Methods

#### Participants

Participants included 30 youth with DS recruited from two sites [University of Arizona (*n* = 26) and the National Institute of Mental Health (NIMH; *n* = 4)] and 30 youth with sex chromosome trisomy from the NIMH. Additionally, 30 TD youth from NIMH served as control participants. All participants were matched on chronological age and maternal education levels. We chose to match participants on chronological age (and not mental age as is often done in studies of youth with intellectual disability) because one of the primary goals of the larger investigation was to examine age effects on EF difficulties. Thus, we needed to match groups on age so that we could examine how the DS or +1X groups deviated from typically developing peers of the same age. To control for IQ differences among the groups, follow-up analyses were completed with nonverbal IQ covaried, as described further below. We also matched groups on maternal education levels in order to compare youth from similar family backgrounds (i.e., families with similar levels of educational achievement).

Participants with DS were recruited through family support groups local to the two sites and nationally. Participants were included in the current study if they had a confirmed medical diagnosis of DS according to parent report and had a complete BRIEF rating form (school age version).

Participants with sex chromosome trisomy (XXY and XXX) were recruited nationally with the help of parent advocacy groups to participate in a larger study of cognitive and brain development in youth with sex chromosome aneuploidies being conducted at the NIMH. The current sample represented a subsample of the larger group included in the NIMH study. To be included in the current sample, participants need to have a complete BRIEF form and also have a prenatal diagnosis of either Trisomy X syndrome or Klinefelter syndrome. This additional inclusion criterion was imposed on the sex chromosome trisomy group and not the DS group because unlike DS, many individuals with sex chromosome trisomies are unaware of their diagnosis (Boyd et al., 2010). Because there are not consistent physical dysmorphologies associated with the addition of an X-chromosome, many individuals go undiagnosed. As a result, samples that include postnatally-identified participants may be prone to include children with higher rates of learning and behavioral difficulties. This is believed to be the case, because often it is the presence of learning or behavioral difficulties that leads professionals to complete genetic testing in the absence of frank physical dysmorphologies. Thus, by excluding postnatally-diagnosed participants with +1X, we sought to provide a description of EF difficulties in this group that are not overly biased by participants who are having behavioral difficulties (that consequently led to the genetic testing and diagnosis). As a result, our descriptions of EF difficulties in this group may be more conservative than if we had included those with postnatal diagnoses. However, we deemed this as preferable to overstating the EF difficulties associated with sex chromosome trisomy.

TD participants were recruited through advertisements in the community and nationally. Prior to enrollment in the study, parents were interviewed about their child's development. Only participants without a history of developmental, learning, or psychiatric difficulties were included in the TD group.

For participants over the age of majority and with cognitive capacity to consent independently, written consent was obtained from the participant. For minors and those without capacity to consent independently, written consent was obtained from parents or legal guardians and the participant provided assent. The three studies included in this paper were reviewed and approved by the Institutional Review Board of each participating institution.

Demographic information about the three groups including age, sex, race, nonverbal IQ, and maternal education is summarized in Table 1. As shown in the table, groups did not differ on any of the demographic variables except for nonverbal IQ, which was expected. As will be seen in the Results section, IQ differences among the groups were controlled statistically in follow-up analyses and their effects on the study's findings are discussed.

**Table 1:** **Demographic information about the Down syndrome (DS), Sex Chromosome Trisomy (XXY & XXX; +1X), and Typically Developing (TD) control groups**.

	**DS (*n* = 30)**			**+1X (*n* = 30)**			**TD (*n* = 30)**			
	***M***	***SD***	**Range**	***M***	***SD***	**Range**	***M***	***SD***	**Range**	**F or *X*^2^**
Chron. Age	11.34	3.02	7–17	11.61	3.29	5–18	11.28	2.69	6–17	*F*_(2, 87)_ < 1, *p* > 0.9
Nonverbal IQ^∧^	52.41	13.2	40–87	100.75	15.70	74–135	110.37	11.97	86–139	*F*_(2, 84)_ = 150.56, *p* < 0.001
Maternal Ed.	15.68	2.17	11–21	15.57	1.79	12–19	16.13	2.26	12–21	*F*_(2, 87)_ < 1, *p* > 0.5
	***n***	**%**		***n***	**%**		***n***	**%**		
Sex—male	15	50		15	50		15	50		*X*^2^ < 1, *p* > 0.9
Race/Ethnicity—WNH	20	67		26	87		25	83		*X*^2(2)^ = 4.1, *p* > 0.12

*^∧^DS group n = 29 with Nonverbal IQ data; +1X group, n = 28 with Nonverbal data; missing data on 3 participants total for Nonverbal IQ. Chron. Age, Chronological Age; Maternal Ed, Year of Maternal Education; WNH, White, Non-hispanic*.

#### Measures

##### Everyday executive function skill assessment

Parents of participants completed the school-age BRIEF form, developed for youth ages 5–18 years (Gioia et al., 2000). The school-age BRIEF has been utilized effectively in studies of DS, and in the validation of the Arizona Cognitive Test Battery for DS, the measure correlated with laboratory tasks of EF and memory (e.g., CANTAB; Edgin et al., 2010). It has also been used effectively in studies of youth with sex chromosome aneuploidies (Janusz et al., 2011; Samango-Sprouse et al., 2015).

The BRIEF is an 86-item questionnaire that assesses EF behaviors in various domains. Caregivers describe their child's behavior using a 3-point Likert scale indicating how frequently their child engages in a given behavior (never = 1, sometimes = 2, often = 3). Higher scores denote greater problems. The BRIEF includes eight clinical scales: Inhibit, Shift, Emotional Control, Initiate, Working Memory, Plan/Organize, Organization of Materials, and Monitor. These scales were both theoretically and empirically derived. They are combined to create two indices: the Behavioral Regulation Index (Inhibition + Shift + Emotional Control Scales) and the Metacognition Index (Initiate + Working Memory + Plan/Organize + Organization of Materials + Monitor Scales). For the current investigation, T-scores were utilized to compare scores on the different scales across the groups. These T-scores were derived from the BRIEF manual. They are age and sex-adjusted and have a mean of 50; higher T-scores denote greater difficulties. Descriptions of the eight clinical scales are provided in Table 2.

**Table 2:** **Descriptions of BRIEF Clinical Scales belonging to the Behavioral Regulation and Metacognition Indices**.

**Scale name**	**Index**	**Description**	**Item examples**
Inhibit	BR	Evaluates behaviors related to the ability to inhibit an impulse and stop behaviors when appropriate	Being fidgety or impulsive; getting more out of control than same-age peers
Shift	BR	Includes behaviors related to the ability to move from situation to situation or shift set	Resisting change in routines; becoming upset in new situations
Emotional control	BR	Evaluates behaviors related to the modulation of emotions	Having angry outbursts and getting upset easily
Working memory	MC	Assesses behaviors related to holding information online in memory in order to complete tasks with greater than one step	Having difficulty remembering multiple things to do or completing tasks with more than one step; having a short attention span
Plan/Organize	MC	Evaluates behaviors related to anticipating future events and organizing information and behavior to complete a goal	Having difficulty finding belongings, getting through routines, or initiating tasks
Initiate	MC	Examines generative behavior—i.e., beginning tasks and thinking of ideas/responses	Having difficulty getting tasks started; taking initiative
Organization of materials	MC	Measures how an individual organizes personal spaces and belongings	Leaving areas messy; having difficulties finding belongings

*BR, Behavior Regulation; MC, Metacognition*.

While the BRIEF was not created to test differences in cool vs. hot EF difficulties, we believe that this is a useful classification system for two reasons. First, the BRIEF's two indices map roughly onto these constructs (e.g., the Metacognition Index measures common cool EF skills, including working memory and planning, while the Behavior Regulation index measures common hot EF skills, such as emotional control and inhibition). Second, this classification system has been a useful way to conceptualize the nature of EF difficulties in DS in our past work, albeit with the preschool BRIEF (Lee et al., 2011a; Daunhauer et al., 2014).

##### Nonverbal intelligence testing

Participants included in this study completed the Kaufman Brief Intelligence Test—Second Edition (*n* = 26; Kaufman and Kaufman, 2004), the Differential Ability Scales—Second Edition (*n* = 4; Elliott, 2007), or the Wechsler Abbreviated Scale of Intelligence Test (*n* = 30; Wechsler, 1999) per individual study protocols. We report on Nonverbal IQ rather than Full Scale (or Verbal) IQ in this study, because not all participants in Study 3 (which includes an independent sample of participants) completed an IQ test with a verbal portion. Thus, for consistency in reporting across the studies, we report only nonverbal IQ scores here. However, when using an estimate of overall intellectual ability as a covariate in analyses, we report the findings for nonverbal IQ but also note if they hold when Full Scale IQ is used as a covariate instead.

#### Statistical analyses

Prior to completing primary analyses, the effects of sex on the eight BRIEF scales were evaluated to determine if it needed to be included in our models. This was done by running a series of independent samples *t*-tests within the three groups and comparing scores for males and females. No statistically significant sex differences were detected once the false discovery rate (FDR; Hochberg and Benjamini, 1990) correction was applied. The only sex differences that approached significance were found within the DS group for the Shift scale (where males had greater difficulties; *p* = 0.02) and the TD group on the Organization of Materials scale (where females had greater difficulties; *p* = 0.04).

Because these differences did not exceed thresholds for statistical significance, sex was excluded from the models and primary analyses were completed as follows. To examine differences in BRIEF profiles for the DS and +1X groups (as compared to TD controls), a series of mixed-model ANOVAs was completed. First, a 3 × 2 mixed-model ANOVA was completed with one between-subject factor (Group: DS vs. +1X vs. TD) and one within-subject factor (BRIEF Index: Behavior Regulation vs. Metacognition). This was followed by an additional mixed measure ANOVA in which the eight scales that constitute the Behavior Regulation and Metacognition indices were compared across groups. These ANOVAs were followed by tests of simple effects (using *t*-tests) when necessary and FDR correction was applied to adjust for multiple comparisons. Lastly, to account for differences in nonverbal IQ among the groups, ANCOVAs were run with nonverbal IQ covaried.

### Results

#### Do youth with DS and +1X have distinct profiles on the BRIEF?

The 3 × 2 mixed-model ANOVA with one between-subject factor (Group: DS vs. +1X vs. TD) and one within-subject factor (BRIEF Index: Behavior Regulation vs. Metacognition) revealed a main effect of index [*F*_(1, 87)_ = 9.04, *p* < 0.004], a main effect of group [*F*_(2, 87)_ = 22.37, *p* < 0.001], but no group × index interaction [*F*_(2, 87)_ = 2.04, *p* > 0.13].

The main effect of index was such that scores tended to be lower (denoting fewer difficulties) on the Behavior Regulation Index than Metacognition Index overall. (However, it is important to note that this main effect appeared to be driven by the DS and TD groups, and not the +1X group. Specifically, when paired samples *t*-tests were run comparing the two indices for the three groups separately, the results were significant for the DS and TD groups (ps < 0.01) such that fewer problems with Behavioral Regulation were noted. In contrast, for the +1X group, these scores did not differ (*p* = 0.9), suggesting similar levels of impairment.)

**Figure 1:**
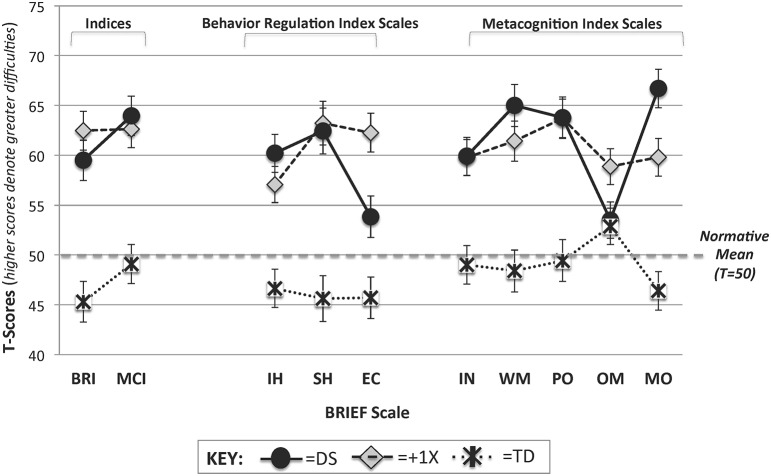
**BRIEF profiles for the DS, +1X, and TD groups**. T-Scores on the BRIEF Indices and Scales are provided for the DS (solid black circles with solid line), +1X (gray diamonds with long dotted line), and TD (asterisks with small dotted line) groups. Note that the normative mean is 50 and that greater T-scores denote greater levels of difficulty. BRI, Behavior Regulation Index; MCI, Metacognition Index; IH, Inhibit; SH, Shift; EC, Emotional Control; IN, Initiate; WM, Working Memory; PO, Plan/Organize; OM, Organization of Materials; MO, Monitor.

The main effect of group was such that the TD controls had lower scores (fewer difficulties) overall than both of the aneuploidy groups (qs < 0.05; FDR corrected for 3 comparisons) which did not differ significantly from one another (*p* > 0.70). These results and those that follow are summarized in Figure 1.

To account for IQ differences among the groups, analyses were re-run with nonverbal IQ covaried. When nonverbal IQ was included as a covariate in a 3 × 2 mixed-model ANCOVA, the main effect of index was no longer significant, but the main effect of group remained [*F*_(2, 83)_ = 13.01, *p* < 0.001]. Tests of simple effects revealed that the TD group continued to outperform the +1X group (*q* < 0.05; FDR corrected for 3 comparisons). However, the DS group's index scores no longer differed from the TD or +1X groups. These analyses were also run with Full Scale IQ covaried and results were largely the same.

Next the eight BRIEF scales were submitted to a 3 × 8 mixed-model ANOVA with one between-subject factor (Group: DS vs. +1X vs. TD) and one within-subject factor (Scale: the eight BRIEF scales). Results revealed a main effect of scale [*F*_(5.06, 440.10)_ = 5.39, *p* < 0.001], a main effect of group [*F*_(2, 87)_ = 21.57, *p* < 0.001] and a group X scale interaction [*F*_(10.12, 440.10)_ = 6.87, *p* < 0.001] (Note: Because the assumption of sphericity was violated, the Greenhouse Geisser adjustment was applied and the degrees of freedom were adjusted).

Tests of simple effects (FDR adjusted for 24 comparisons) revealed that TD controls had lower scores (fewer difficulties) than the +1X group on all of scales (qs < 0.05; FDR corrected). The TD group also differed from the DS group on all scales (qs < 0.05; FDR corrected) except for the Organization of Materials scale (*p* = 0.87). In contrast, for the DS and +1X groups, only two scales differed significantly when FDR correction was applied: Emotional Control (DS < +1X; fewer problems) and Monitor (DS > +1X; more problems). When the unadjusted *p*-values were considered, the DS group had lower scores (denoting fewer difficulties) than the +1X group on the Organization of Materials Scale (*p* = 0.04) as well.

In order to examine the pattern or profile of scores *within* each group, differences in performance on the eight scales were evaluated (and FDR correction was applied for 24 comparisons; 8 for each group). This was done by calculating the group mean on the eight scales and then comparing each scale to this value using paired samples *t*-tests. For the TD group, only the Organization of Materials scale was significantly higher than the overall mean (*q* < 0.05), indicating that this was an area of relative weakness. For the +1X group, there were no significant differences between the individual scales and the overall mean. In contrast, the DS group demonstrated several peaks and valleys in their profile. The following scores were higher than the mean (denoting relative weaknesses): Working Memory and Monitor. In contrast, the following scores were lower than the mean (denoting relative strengths): Emotional Control and Organization of Materials.

In order to control for nonverbal IQ differences among the groups, a 3 × 8 mixed-model ANCOVA was run with nonverbal IQ included as a covariate. With nonverbal IQ in the model, the main effect of group [*F*_(2, 83)_ = 11.96, *p* < 0.001] and the Group X Scale interaction [*F*_(10.38, 430.56)_ = 2.36, *p* < 0.01] remained significant (Note: Because the assumption of sphericity was violated, the Greenhouse Geisser adjustment was applied and the degrees of freedom were adjusted). Tests of simple effects (FDR adjusted for 24 comparisons) revealed that the TD group received lower scores (denoting fewer difficulties) than the +1X group on all scales (qs < 0.05) except for Organization of Materials. For the DS group, no scales differed significantly from the +1X or TD groups with nonverbal IQ included as a covariate in the model. However, when unadjusted *p*-values were considered, the DS group's score on the Emotional Control scale continued to be lower (denoting fewer difficulties) than +1X group (*p* = 0.02) while their scores on the Working Memory (*p* = 0.03), Monitor (*p* = 0.03), and Inhibit (*p* = 0.04) scales were higher than the TD group (denoting greater difficulties). Lastly, when Full Scale IQ was covaried instead of nonverbal IQ, the main effect of group and group x scale interaction remained statistically significant. However, the tests of simple effects revealed slightly different results. While Emotional Control continued to be significantly lower (denoting fewer difficulties) in the DS than the +1X group (uncorrected *p* < 0.05), the differences noted for the DS and TD groups described above were not statistically significant.

### Summary and discussion: Study 1

In this study, we sought to evaluate the specificity of the DS and +1X profiles on the BRIEF by contrasting scores with one another and a TD control group matched on chronological age and maternal education levels. First, to evaluate the profile of differences associated with hot vs. cool EF abilities, we contrasted scores on the Behavior Regulation (which evaluates behaviors that are typically associated with hot EF abilities) and Metacognition (which evaluates behaviors that are typically associated with cool EF abilities) indices of the BRIEF. Replicating our prior findings using the BRIEF-P (Lee et al., 2011a; Daunhauer et al., 2014), we find that participants with DS received higher scores (denoting greater difficulty) on the Metacognition Index than the Behavior Regulation Index of the BRIEF, consistent with greater cool EF difficulties. However, this pattern of scores was not specific to DS, but rather was similar to what was found in the TD group. While there was no group x condition interaction for this analysis, it is important to note that the pattern of index scores for DS was different than the pattern found for the +1X group. Specifically, there was no significant difference between the two indices for this group (*p* = 0.9), suggesting similar levels of difficulties in these two EF domains for youth with +1X.

When the eight scales were compared for the groups of youth with DS and +1X, evidence for both shared and unique features were found. Regarding the shared features, the DS and +1X groups demonstrated similar degrees of EF difficulty on the following scales (all of which were elevated relative to TD controls): Inhibit, Shift, Initiate, Working Memory, and Plan/Organize. Additionally, the groups did not differ on Organization of Materials scale; however, the +1X group's scores were elevated relative to TD controls, while the DS and TD control scores did not differ (*p* = 0.87).

With regard to differences/unique features, the DS group demonstrated greater levels of impairment than +1X group on the Monitor scale; the opposite was true for the Emotional Control scale where the +1X group demonstrated greater levels of impairment. In addition to these two differences, the greatest evidence for *specificity* of BRIEF profiles for the +1X and DS groups came from an examination of the pattern of scores across the BRIEF scales. While the +1X group had a relatively flat profile of scores on the BRIEF (denoting similar levels of difficulties on the different scales), the DS group demonstrated a much more variable profile. Specifically, weaknesses were noted on the Working Memory and Monitor scales while strengths were noted on the Emotional Control and Organization of Materials scales.

## Study 2: Contrasting age-effects on BRIEF scales for the DS +1X groups

In this cross-sectional study, we examined the relations between age and EF difficulties in youth with DS and those with +1X. In particular, we sought to test hypotheses about the stability or variability in the severity of EF difficulties for youth with DS and +1X relative to youth with TD.

### Methods

#### Participants

Participants were the same as those included in Study 1. See Method section above and Table 1 for details.

#### Measures

##### Everyday executive function skill assessment

Again, the BRIEF was used. However, unlike Study 1, raw scores on the BRIEF scales were used as dependent variables rather than age- and sex-adjusted T-scores. Raw scores were preferred over T-scores so that relations between age and total difficulties (unadjusted for age) could be evaluated. In order to allow easy comparison across scales, mean item severity scores were calculated for the eight scales. Specifically, scores on the items included in each scale were totaled and were divided by the number of items in that scale.

#### Statistical analyses

To examine age-related differences in scores among the DS, +1X and TD groups, hierarchical linear regression was used, with three steps: (1) age, (2) group, and (3) age X group interaction. This last step was used to evaluate whether relations between age and EF difficulties varied among the three groups. If this last step was significant, then Pearson correlation coefficients between age and raw scores for each of the pairs (TD vs. DS; TD vs. +1X; +1X vs. DS) were contrasted using a Fishers-R-to-Z transformation.

### Results

#### Are there similar relations between age and BRIEF scale ratings for youth with DS, +1X, and those with typical development?

**Figure 2:**
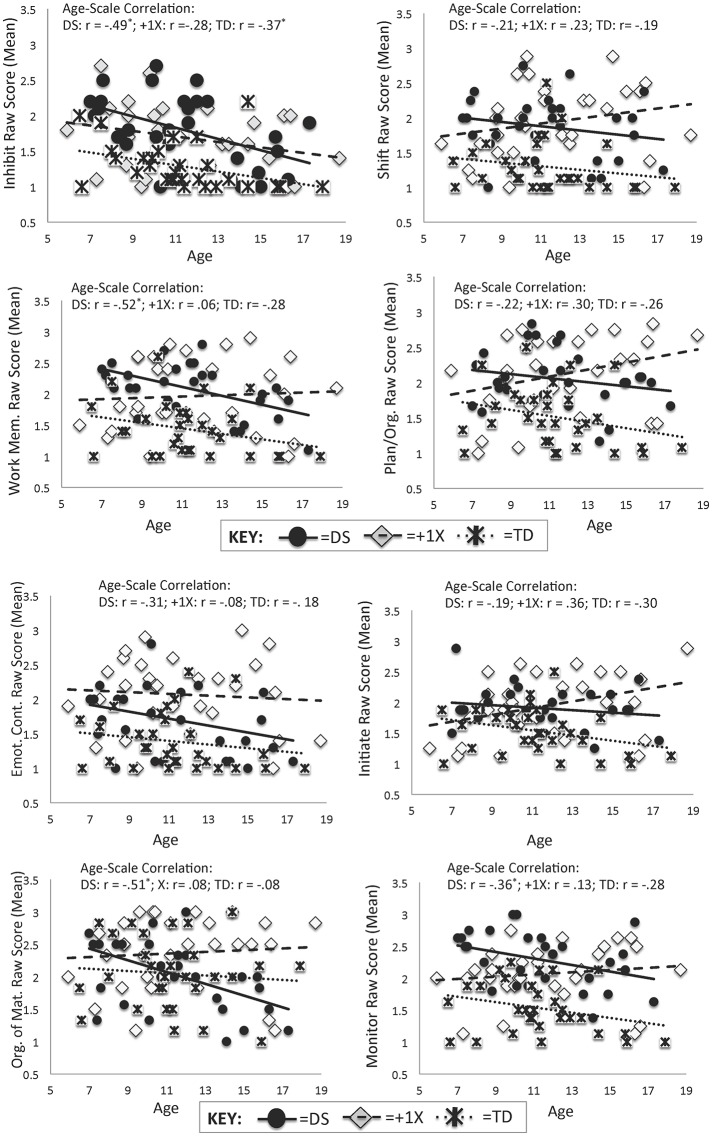
**Scatterplots for Age and BRIEF Scales by Group**. Scatterplots display the relations between age and raw scores on the BRIEF scales for the DS (solid black circles with solid line), +1X (gray diamonds with long dotted line), and TD (asterisks with small dotted line) groups. Note that higher scores denote greater difficulties.

Results of hierarchical linear regressions evaluating the effects of age, group, and their interaction for the eight clinical scales of the BRIEF are summarized in Table 3 and in Figure 2. For six of the eight scales, the effects of age did not appear to vary as a function of group—i.e., the magnitude of the relationship between BRIEF scale raw mean scores and age was similar for the TD, DS, and +1X groups. However, for two of the scales—Initiate and Plan/Organize—there were age X group interactions in the prediction of scores. In both cases, the +1X group's difficulties on the BRIEF appeared to be more severe at older ages while the DS and TD groups' EF difficulties were less severe at older ages (Fisher's *Z* ≥ 1.96, ps < 0.05).

**Table 3:** Hierarchical Linear Regression Results Using Age, Group, and the Age^*^Group Interaction to Predict BRIEF Scale Raw Scores (Means).

**DV**	**Step**	**IVs**	***R***	***R*^2^ Change**	***F* change**	**Df**	***p***
Inhibit	1	Age	0.32	0.11	10.34	1, 88	0.00
	2	Group	0.48	0.12	13.96	1, 87	0.00
	3	Age^*^Group	0.48	0.00	0.04	1, 86	0.84
Shift	1	Age	0.00	0.00	0.00	1, 88	0.98
	2	Group	0.50	0.25	28.90	1, 87	0.00
	3	Age^*^Group	0.52	0.02	2.50	1, 86	0.12
Emotional control	1	Age	0.13	0.02	1.6	1, 88	0.21
	2	Group	0.53	0.26	30.9	1, 87	0.00
	3	Age^*^Group	0.53	0.00	0.20	1, 86	0.66
Initiate	1	Age	0.04	0.00	0.13	1, 88	0.72
	2	Group	0.38	0.14	14.10	1, 87	0.00
	3	Age^*^Group	0.46	0.07	7.64	1, 86	0.01
Working memory	1	Age	0.16	0.03	2.25	1, 88	0.14
	2	Group	0.43	0.16	17.06	1, 87	0.00
	3	Age^*^Group	0.45	0.02	1.64	1, 86	0.20
Plan/Organize	1	Age	0.01	0.00	.01	1, 88	0.93
	2	Group	0.46	0.22	23.82	1, 87	0.00
	3	Age^*^Group	0.51	0.05	5.25	1, 86	0.02
Organization of materials	1	Age	0.15	0.02	1.89	1, 88	0.17
	2	Group	0.28	0.06	5.26	1, 87	0.02
	3	Age^*^Group	0.29	0.01	0.81	1, 86	0.37
Monitor	1	Age	0.10	0.01	1.07	1, 88	0.30
	2	Group	0.44	0.18	19.45	1, 87	0.00
	3	Age^*^Group	0.46	0.02	1.94	1, 86	0.17

### Summary and discussion: Study 2

In the current cross-sectional study, we evaluated the relations between age and EF performance on the BRIEF in youth with DS, +1X, and those with TD. Largely, there was support for similar relations between age and scale scores for the DS and TD groups, lending support for the developmental stability hypothesis for the DS group. As can be seen in Figure 2, the trend in the data for the DS group was for fewer difficulties with increasing age. This paralleled the findings in the TD group. Given the small sample size and the fact that the developmental stability hypothesis is essentially supporting the null hypothesis, Study 3 was completed with a larger independent sample to determine if these results could be replicated across a slightly larger age range (ages 4–24 years) and with a larger group.

For the +1X group, the findings were mixed—for six of the scales, there was support for developmental stability. However, for the Initiate and Plan/Organize scales, there appeared to be support for developmental variability. In both cases, the trend in the data was for caregivers' ratings of EF difficulties to increase with increasing age (denoting greater difficulties later). However, given the cross-sectional nature of these data as well as the small sample size, these findings must be interpreted cautiously. Further discussion and interpretation of these findings will be provided in the General Discussion section.

## Study 3: A replication study of age-BRIEF relations in the DS and TD groups

In this cross-sectional study, we sought to replicate our earlier findings of developmental stability on the BRIEF with a larger independent sample of youth with DS (*n* = 85) and a TD control group (*n* = 43).

### Methods

#### Participants

Participants included 85 youth with DS recruited from three sites: the University of Arizona (*n* = 36), Colorado State University (*n* = 31), and NIMH (*n* = 18). A total of 43 typically developing control participants matched on chronological age and maternal education levels were recruited from two sites: the University of Arizona (*n* = 13) and the National Institute of Mental Health (*n* = 30). Rationale for matching on chronological age (rather than mental age) can be found in the Method section of Study 1.

Demographic information about the two groups including age, sex, race, nonverbal IQ, and maternal education is summarized in Table 4. As shown in the table, groups did not differ on any of the demographic variables except for nonverbal IQ, which was expected and similar to the findings from the previous two studies.

**Table 4:** **Demographic information about the Down syndrome (DS) and typically developing (TD) control groups**.

	**DS (*n* = 85)**			**TD (*n* = 43)**			
	***M***	***SD***	**Range**	***M***	***SD***	**Range**	***X*^2^ or T-stat**
Chron. Age	12.3	5.03	4–24	12.35	5.41	4–22	*t*_(126)_ < 1, *p* > 0.95
Nonverbal IQ^∧^	52.53	14.72	24–112	106.4	12.22	67–131	*t*_(124)_ = 20.59, *p* < 0.001
Maternal Ed.	15.69	2.34	6–21+	16.02	2.10	13–21+	*t*_(186)_ < 1, *p* > 0.43
	***n***	**%**		***n***	**%**		
Sex—male	48	57		20	47		*X*^2^ < 1.2, *p* > 0.28
Race/Ethnicity—WNH^*^	52	63		28	65		*X*^2^ < 1, *p* > 0.85

*^∧^DS group n = 83, missing data on 2 participants*.

* *DS group n = 82, missing complete race and ethnicity information on 3 participants*.

*Chron. Age, Chronological Age; Maternal Ed = Year of Maternal Education; WNH, White Non-hispanic*.

#### Measures

##### Everyday executive function skill assessment

Unlike the prior study in which the school-age BRIEF was utilized, the current study included participants with either the school-age BRIEF, developed for participants age 5–18 or the preschool BRIEF (BRIEF-P), developed for participants age 2–5. The inclusion of the two versions of the BRIEF permitted combining data collected on participants with DS over a large age range who participated in studies at the three sites listed above.

Despite differences in targeted age range, there are a number of shared items on the BRIEF and BRIEF-P that permitted the creation of composite scores that could be used regardless of the version of the BRIEF that was administered. As will be described in further detail below (under the subheading, “Creation of Study-derived BRIEF Composites”), 41 items were extracted from the two versions of the BRIEF to create five composite scores that mapped onto the five indices included on the preschool BRIEF—the Emotional Control, Inhibit, Shift, Working Memory, and Plan/Organize indices.

The version of the BRIEF administered was determined by site protocol. For participants with DS recruited at Colorado State University and the National Institute of Mental Health, mental age was used to determine the version of the BRIEF that was administered, consistent with prior publications from our labs (Lee et al., 2011a; Daunhauer et al., 2014). Thus, even if participants were >5 years of age, they were given the preschool BRIEF if their mental age was between the ages of 2 and 5 years. Similarly, if they were greater than 18 years of age but their mental age was between the ages of 5 and 18, they were given the school-age BRIEF. Participants with DS recruited from the University of Arizona and all but one control participant (who was over the age of 18) were given the chronological age appropriate version of the BRIEF. While the correct version of the BRIEF for the one typically developing participant over the age of 18 (age 22 years) was technically the BRIEF-A (adult), no one else in the study had data on the BRIEF-A. Thus, we asked that this adult request that his/her parents complete the school age BRIEF (given that we were only using raw scores on certain items for this particular study, as described below).

In total, 44 participants with DS and 30 TD controls received the BRIEF; 41 participants with DS and 13 TD controls received the BRIEF-P. As described earlier, the BRIEF has 86 items; the BRIEF-P has 63 items. Both versions use the same 3-point Likert scale with which caregivers indicate how frequently their child engages in a given behavior (never = 1, sometimes = 2, often = 3). Higher scores denote greater problems on both instruments.

Unlike the BRIEF which includes eight clinical scales (see Table 2 for details), the BRIEF-P includes five clinical scales that are a subset of the eight from the BRIEF. These include Inhibit, Shift, Emotional Control, Working Memory, and Plan/Organize. These scales were also theoretically and empirically derived. They are combined to form three indices: the Inhibitory Self-Control Index (Inhibition + Emotional Control Scales), Flexibility Index (Shift + Emotional Control Scales), and Emergent Metacognition Index (Working Memory + Plan/Organize Scales).

##### Creation of study-derived BRIEF composites

In order to examine age effects using the two instruments, shared items from the BRIEF and BRIEF-P were extracted for each participant and composites were created. Because there are fewer clinical scales on BRIEF-P and all five of its scales are also found on the BRIEF (which has three additional scales), item composites were created based upon the item's scale on the BRIEF-P (i.e., if an item was a part of the Working Memory scale on the BRIEF-P, it was included in the Working Memory composite in this scheme). Thus, the current study included five composites, which mapped onto the five clinical scales from the BRIEF-P: Inhibit, Shift, Emotional Control, Working Memory, and Plan/Organize. See Table 2 for descriptions of the types of items that are included in these five scales.

Scores for the five composites were created by calculating the average item rating across all items included in that composite. Items were included in the composites created here if they were identical on the BRIEF and BRIEF-P or if the content of the words varied slightly but the targeted behavior was the same. For example, the BRIEF item “has to be closely supervised” was considered equivalent to the BRIEF-P item “has to be more closely supervised than similar playmates.” Similarly, the BRIEF item “is fidgety,” was considered equivalent to the BRIEF-P item “is fidgety, restless, or squirmy.” In total, 41 items were extracted from the two instruments. Twenty had identical wording and 21 had similar wording.

The BRIEF and BRIEF-P items that were included in the five composites created for this investigation are summarized below. The BRIEF item is listed first, followed by the BRIEF-P item (which has a P with it).

The *Emotional Control Composite* included items: 1 & 1P, 7 & 6P, 25 & 16P, 26 & 21P, 45 & 36P, 62 & 31P, 64 & 26P, 70 & 11P.

The *Inhibit Composite* included items: 34 & 3P, 38 & 18P, 42 & 33P, 44 & 43P, 78 & 13P, 54 & 52P, 55 & 54P, 59 & 60P, 63 & 38P, 81 & 23P, 82 & 28P.

The *Shift Composite* included items: 6 & 5P, 12 & 15P, 23 & 45P, 80 & 35P.

The *Plan/Organize composite* included items: 10 & 9P, 28 & 39P, 33 & 14P, 67 & 44P, 69 & 34P, 75 & 19P, 86 & 24P.

The *Working Memory Composite* included items: 2 & 2P, 9 & 61P, 17 & 12P, 21 & 22P, 24 & 27P, 27 & 32P, 32 & 37P, 37 & 42, 47 & 51P, 57 & 59P, 83 & 47P.

To demonstrate the similarities in the composite created for this study and the raw score for the corresponding clinical scale on the BRIEF or BRIEF-P, Pearson correlation coefficients were run. For the BRIEF, the correlations between the study-generated composites and raw totals were as follows for the Inhibit, Shift, Emotional Control, Working Memory, and Plan/Organize scales, respectively: 0.94, 0.94, 0.99, 0.98, 0.75. For the BRIEF-P, the correlations were as follows, respectively: 0.99, 0.94, 0.99, 0.97, 0.96.

##### Nonverbal intelligence testing

Participants at the three sites were given different intelligence tests per individual study protocols. These included the Leiter International Performance Scale—Revised (*n* = 31; Roid and Miller, 1997), the Kaufman Brief Intelligence Test—Second Edition (*n* = 55; Kaufman and Kaufman, 2004), the Differential Ability Scales—Second Edition (*n* = 12; Elliott, 2007), the Wechsler Abbreviated Scale of Intelligence (*n* = 29; Wechsler, 1999), and the Wechsler Preschool and Primary Scale of Intelligence—Third Edition (*n* = 1; Wechsler, 2002).

#### Statistical analyses

Similar to Study 2, age effects on the five BRIEF composites were examined using hierarchical linear regression with three steps: (1) age, (2) group, and (3) age X group interaction. This last step was the step used to evaluate whether relations between age and EF difficulties varied for the DS and TD groups.

### Results

Results of regression analyses can be found in Table 5. As can be seen, there were no age X group interactions for any of the regression equations, consistent with findings of Study 2. Rather, relations between age and BRIEF scores were similar for the DS and TD groups.

**Table 5:** Hierarchical linear regression results using Age, Group, and the Age^*^Group interaction to predict BRIEF scale raw scores (Means).

**DV**	**Step**	**IVs**	***R***	***R*^2^ Change**	**F change**	**df**	***p***
Inhibit Composite	1	Age	0.24	0.06	7.65	1, 126	0.01
	2	Group	0.58	0.28	51.93	1, 125	0.00
	3	Age^*^Group	0.58	0.00	0.09	1, 124	0.76
Shift Composite	1	Age	0.01	0.00	0.01	1, 126	0.93
	2	Group	0.44	0.19	29.29	1, 125	0.00
	3	Age^*^Group	0.44	0.00	0.56	1, 124	0.46
Emotional Control Composite	1	Age	0.14	0.02	2.36	1, 126	0.13
	2	Group	0.31	0.08	10.66	1, 125	0.00
	3	Age^*^Group	0.32	0.01	0.66	1, 124	0.42
Work. Mem. Composite	1	Age	0.09	0.01	1.06	1, 126	0.31
	2	Group	0.67	0.44	99.12	1, 125	0.00
	3	Age^*^Group	0.67	0.00	0.15	1, 124	0.70
Plan/Organize Composite	1	Age	0.01	0.00	0.01	1, 126	0.93
	2	Group	0.51	0.26	44.62	1, 125	0.00
	3	Age^*^Group	0.52	0.00	0.39	1, 124	0.54

### Summary and discussion: Study 3

Taken together, the results of Study 3 provide additional support for stability in the DS profile on the BRIEF from early childhood to young adulthood. Specifically, a similar relationship between age and the BRIEF EF composite scores was found for the DS and control groups. For all composites except the Inhibit composite, age effects were non-significant and there were no age X group interactions, indicating that neither group's BRIEF scores were strongly predicted by age. For the Inhibit composite, significant age effects were found, such that inhibit scores improved (decreased) with age. However, these findings were similar in the DS and control groups, as evidenced by the lack of a group X age interaction. Despite the lack of an interaction effect, it is worth noting that the DS group's higher scores on the Inhibit scale paired with parallel rates of decreasing difficulties as compared to controls suggests that these difficulties may continue to lessen into the mid 20s to early 30s and eventually reach the level of the TD group, albeit at a much older age. This hypothesis would need to be confirmed with an older and/or longitudinal sample.

## General discussion

In this paper, we asked two primary questions: (1) Are there unique EF profiles on the BRIEF for school-age children and adolescents (ages 5–18) with DS and those with +1X? (2) Do the relations between age and EF abilities in DS and +1X deviate from what is seen in TD?

With regard to the first question, that of syndromic specificity of BRIEF profiles, we find some evidence for specificity and some for overlap. Specifically, there were several scales on the BRIEF in which the DS and +1X groups were similarly impaired. These included the Inhibit, Shift, Initiate, Working Memory, Plan/Organize, and Organization of Materials scales. In contrast, the DS group received lower scores (denoting fewer difficulties) on the Emotional Control scale while the +1X group received lower scores on the Monitor scale.

Interestingly, the greatest difference between the two groups appears to be in the pattern or profile of scores rather than the absolute values of the scores. More specifically, the +1X group showed a relatively flat profile of scores on the BRIEF—that is, there was little variation in scores across the eight BRIEF scales. In contrast, the DS group had several peaks and valleys in their scores. In particular, scores on the Working Memory and Monitor scales were peaks, denoting greater difficulties, while the Organization of Materials and Emotional Control scales were valleys, denoting relative strengths. These two relative strengths are noteworthy.

First, the finding of relatively lower levels of difficulty with Emotional Control fits with studies suggesting that youth with DS have lower rates of psychiatric difficulties than youth with other developmental disabilities (Dykens, 2007). This also fits with our prior studies suggesting that youth with DS have fewer hot than cool EF difficulties (Lee et al., 2011a; Daunhauer et al., 2014). However, it is important to note that while this is a relative strength in DS, it is not an absolute strength. Rather, difficulties with emotional control are higher in DS than those found in same age typically developing peers (analogous to rates of psychiatric difficulties). Second, relatively lower difficulties on the Organization of Materials scale (which were essentially commensurate with the TD group) may relate to anecdotal reports suggesting that some people with DS are very concerned with the organization of their belongings and prefer to have things be “just so.” We have observed this clinically and have had parents mention that their children can be insistent on the order/organization of particular things in their homes. Despite the speculative nature of these observations, it may be helpful to emphasize that this particular set of skills should be viewed as a relative strength and thus may prove useful in designing interventions aimed at improving organization and planning as it relates to cognitively demanding academic tasks. This is particularly relevant for DS, as two of their greatest weaknesses on the BRIEF were on the Working Memory and Monitor scales. Both of these scales assess abilities that are important for academic outcomes, and thus, developing strategies to improve these skills may be a target for future investigations.

For youth with +1X, executive difficulties appear to be quite significant and uniform. It is noteworthy that this group's mean nonverbal IQ score was over three standard deviations higher than the DS group's, but the group's scores on seven of the eight BRIEF scales were similarly or more impaired. Furthermore, when nonverbal IQ was controlled for in ANCOVA analyses, group differences between the +1X and TD groups remained on seven of the eight scales (with FDR correction for multiple comparisons). This was not the case for the DS group. Thus, it appears that many of the everyday EF difficulties that accompany +1X are well in excess of IQ reductions associated with syndrome.

Furthermore, unlike youth with DS, difficulties with emotional control appear to be related to Klinefelter and Trisomy X syndromes, suggesting that future examinations should probe hot executive difficulties in sex chromosome trisomies in greater detail. This finding fits with studies indicating higher rates of mood and attentional difficulties for females and males with sex chromosome trisomies, respectively (Tartaglia et al., 2006, 2010). Thus, the current results highlight the importance of close monitoring of mood and attentional difficulties for youth with Klinefelter and Trisomy X syndromes. This is especially important given that this study included only prenatally diagnosed participants with sex chromosome aneuploidies (a strength of the current research). Had we permitted the inclusion of participants with postnatally diagnosed sex chromosome aneuploidies, we suspect that we would have found even greater levels of difficulties.

With regard to the second question about the developmental stability of EF difficulties in youth with DS and those with +1X, there was consistent support for developmental stability in the DS group and mixed findings in the +1X group. For the DS group, this question was addressed both in studies 2 and 3 with two independent samples. Results were similar across the two studies—namely, the degree of EF difficulties on all domains of the BRIEF examined were similar to that of TD controls across the study's age range (5–18 years in Study 2 and 4–24 years in Study 3).

In the group of youth with +1X, the overall trend in the data supported the developmental stability hypothesis. However, two scales—Initiate and Plan/Organize—were associated with greater deviations from typical peers (and DS peers) with increased age. These findings must be interpreted very cautiously for several reasons. First and most importantly, this is a cross-sectional study. Thus, we cannot suggest that these skills are worsening over time. There could be a bias in our sample such that more impaired youth tend to be older. However, this seems unlikely given that not all scales were associated with greater difficulties at older ages. To control for any possible IQ confounds in our sample (i.e., a possible confound in which older participants had lower IQ scores), partial correlation analyses were run between each of the BRIEF scales and age with the effects of nonverbal IQ removed. For the three groups, the direction of the relations between age and BRIEF raw scores remained the same (positive correlations between age and both Initiate and Plan/Organize scores in the +1X group and negative correlations for the DS and TD groups).

Second, increases in perceived problems may relate to increased expectations that parents place on older youth with +1X that are not placed on older youth with DS, for example, possibly due to the IQ differences between the groups. It may be that as youth with +1X age, expectations increase and parent ratings reflect this. Future research investigating EF difficulties with laboratory tests may help rule out or confirm this possibility.

Existing research on the relations between age and everyday EF difficulties in other developmental disabilities is limited. One set of investigators (Rosenthal et al., 2013) examined these relations in a cross-sectional sample of youth with autism spectrum disorders and reported a worsening of EF difficulties on the Working Memory, Initiate, and Organization of Materials scales with age (using BRIEF norms and a cross-sectional sample). These findings fit with those found for +1X and contrast with those found for the DS group.

Our findings of stability in the degree of EF difficulties over the course of childhood and into young adulthood in DS may be a specific feature of the behavioral phenotype over the age range studied. This will need to be examined in future research with longitudinal samples. Additionally, it will be important to examine the stability of EF scores on the BRIEF (and using laboratory instruments) across the lifespan in DS, as the heightened rates of precocious Alzheimer's in DS suggest that the fifth and sixth decades of life are times in which EF difficulties may change for some individuals with DS. Furthermore, more research is needed prior to school age to understand the development of EF difficulties from infancy through the preschool years. Thus, it will be crucial to include these age groups in future research.

We now turn to discussing the limitations of our studies. For Study 1, we were limited by small sample sizes. Thus, we may have been underpowered to detect more subtle differences between the DS and +1X groups on the BRIEF scales. Furthermore, given our small samples, we were not able to thoroughly investigate possible sex differences within the groups. While our preliminary investigation of this suggested no large sex differences in male and female scores between the groups, the small samples may have resulted in our being underpowered to detect these differences. We were most concerned about the impact of sex differences within the +1X group, given that males with Klinefelter syndrome and females with Trisomy X syndrome are often considered separately in the literature. However, the overall trend in the BRIEF data examined here was for very similar scores on the BRIEF scales for males and females with +1X. This is consistent with our findings from an earlier study of language difficulties in this population (Lee et al., 2012). Lastly, an additional limitation of Study 1 is that the IQ scores for the DS and +1X groups were markedly different (and different from that of the TD controls). While this likely contributed to differences in performance on the BRIEF, it is important to note that the biggest difference in EF for these two groups appeared when the profile of EF difficulties on the BRIEF was examined *within* each group (i.e., when each BRIEF scale score was compared to the mean of the scale scores for that group). Thus, this set of analyses was not concerned with absolute differences between the groups but rather the profile of scores within each group. Moreover, given the IQ differences between the DS and +1X groups, it is especially noteworthy that the +1X group had EF difficulties that were similar to (or even exceeded) the DS group. This finding should underscore the degree of EF difficulties encountered by youth with Klinefelter and Trisomy X syndromes and encourage future research on the nature of these EF difficulties.

For Studies 2 and 3, the greatest limitation was the cross-sectional research design. We recognize this weakness, but see these studies as first steps forward in describing trajectories of everyday EF difficulties in DS and +1X. While our +1X sample was relatively small, our sample of youth with DS is one of the largest studied with this well-known and validated measure. Thus, these results add to our understanding of the EF profile and possible developmental trends in both DS and +1X.

Clearly, longitudinal studies are needed and should follow this study to confirm (or refute) these findings. Moreover, as stated earlier, it will be important to examine the stability of EF scores (both on the BRIEF and using laboratory measures) across the lifespan in DS and +1X, both at earlier stages in development and later in life. For the DS group, studies of EF early in development would be crucial, given that research suggests declines in intellectual functioning prior to the age of 4 (see Carr, 2012 for a review). Understanding how EF abilities develop during this period could provide important clues to this decline. In addition, studying EF abilities in middle adulthood may provide important predictive information regarding which individuals with DS will go on to develop precocious-onset Alzheimer's disease.

In the +1X group, further research is needed to examine systematically possible changes in EF skills over time. While early prospective studies of individuals with sex chromosome aneuploidies from the 1980s set the stage for a lifespan perspective on the development of these disorders (see Bender and Berch, 1987 for a review), those studies were characterized by small sample sizes. Thus, additional research is needed that examines outcomes longitudinally with larger groups and additional outcome measures.

## Author contributions

NL contributed to study design, analyzed, and interpreted data, and wrote the manuscript; PA, EW, EA, LC, and JB contributed substantially to data acquisition and provided critical revisions of the manuscript; JG, LD, DF, and JE contributed to study design and conceptualization, interpretation of data, critically revising the manuscript.

### Conflict of interest statement

The authors declare that the research was conducted in the absence of any commercial or financial relationships that could be construed as a potential conflict of interest.

## Abbreviations

EF: Executive Function
DS: Down syndrome
+1X: Participants with an additional X-chromosome (Klinefelter syndrome in males and Trisomy X syndrome in females).
